# Evaluating palliative care case conferences in primary care for patients with advanced non-malignant chronic conditions: a cluster-randomised controlled trial (KOPAL)

**DOI:** 10.1093/ageing/afae100

**Published:** 2024-05-23

**Authors:** Tina Mallon, Josefine Schulze, Judith Dams, Jan Weber, Thomas Asendorf, Silke Böttcher, Uta Sekanina, Franziska Schade, Nils Schneider, Michael Freitag, Christiane Müller, Hans-Helmut König, Friedemann Nauck, Tim Friede, Martin Scherer, Gabriella Marx

**Affiliations:** Department of General Practice and Primary Care, University Medical Centre Hamburg-Eppendorf, Martinistraße 52, 20246 Hamburg, Germany; Department of General Practice and Primary Care, University Medical Centre Hamburg-Eppendorf, Martinistraße 52, 20246 Hamburg, Germany; Department of Health Economics and Health Care Research, University Medical Centre Hamburg-Eppendorf, Martinistraße 52, 20246 Hamburg, Germany; Institute for General Practice and Palliative Care, Hannover Medical School, Carl-Neuberg-Straße 1, 30625 Hannover, Germany; Department of Medical Statistics, University Medical Centre Göttingen, Humboldtallee 32, 37073 Göttingen, Germany; Division of General Practice, Carl von Ossietzky University of Oldenburg, Ammerlaender Heerstraße 114-118, 26129 Oldenburg, Germany; Department of General Practice, University Medical Centre Göttingen, Humboldtallee 38, 37073 Göttingen, Germany; Department of Palliative Medicine, University Medical Centre Göttingen, Von-Siebold-Str. 3, 37075 Göttingen and Institute for General Practice and Palliative Care, Hannover Medical School, Carl-Neuberg-Str. 1, 30625 Hannover, Germany; Institute for General Practice and Palliative Care, Hannover Medical School, Carl-Neuberg-Straße 1, 30625 Hannover, Germany; Division of General Practice, Carl von Ossietzky University of Oldenburg, Ammerlaender Heerstraße 114-118, 26129 Oldenburg, Germany; Department of General Practice, University Medical Centre Göttingen, Humboldtallee 38, 37073 Göttingen, Germany; Department of Health Economics and Health Care Research, University Medical Centre Hamburg-Eppendorf, Martinistraße 52, 20246 Hamburg, Germany; Department of Palliative Medicine, University Medical Centre Göttingen, Von-Siebold-Str. 3, 37075 Göttingen, Germany; Department of Medical Statistics, University Medical Centre Göttingen, Humboldtallee 32, 37073 Göttingen, Germany; Department of General Practice and Primary Care, University Medical Centre Hamburg-Eppendorf, Martinistraße 52, 20246 Hamburg, Germany; Department of General Practice and Primary Care, University Medical Centre Hamburg-Eppendorf, Martinistraße 52, 20246 Hamburg, Germany

**Keywords:** palliative care, case conference, interprofessional communication, chronic care, primary health care, older people

## Abstract

**Background:**

Patients with congestive heart failure (CHF), chronic obstructive pulmonary disease (COPD) and dementia are underrepresented in specialist palliative home care (SPHC). However, the complexity of their conditions requires collaboration between general practitioners (GPs) and SPHC teams and timely integration into SPHC to effectively meet their needs.

**Objective:**

To facilitate joint palliative care planning and the timely transfer of patients with advanced chronic non-malignant conditions to SPHC.

**Methods:**

A two-arm, unblinded, cluster-randomised controlled trial. 49 GP practices in northern Germany were randomised using web-based block randomisation. We included patients with advanced CHF, COPD and/or dementia. The KOPAL intervention consisted of a SPHC nurse–patient consultation followed by an interprofessional telephone case conference between SPHC team and GP. The primary outcome was the number of hospital admissions 48 weeks after baseline. Secondary analyses examined the effects on health-related quality of life and self-rated health status, as measured by the EuroQol 5D scale.

**Results:**

A total of 172 patients were included in the analyses. 80.4% of GP practices had worked with SHPC before, most of them exclusively for cancer patients. At baseline, patients reported a mean EQ-VAS of 48.4, a mean quality of life index (EQ-5D-5L) of 0.63 and an average of 0.80 hospital admissions in the previous year. The intervention did not significantly reduce hospital admissions (incidence rate ratio = 0.79, 95%CI: [0.49, 1.26], *P* = 0.31) or the number of days spent in hospital (incidence rate ratio = 0.65, 95%CI: [0.28, 1.49], *P* = 0.29). There was also no significant effect on quality of life (∆ = −0.02, 95%CI: [−0.09, 0.05], *P* = 0.53) or self-rated health (∆ = −2.48, 95%CI: [−9.95, 4.99], *P* = 0.51).

**Conclusions:**

The study did not show the hypothesised effect on hospitalisations and health-related quality of life. Future research should focus on refining this approach, with particular emphasis on optimising the timing of case conferences and implementing discussed changes to treatment plans, to improve collaboration between GPs and SPHC teams.

## Key Points

- Patients with advanced non-malignant chronic conditions might benefit from an interprofessional care approach, particularly from collaboration between general practitioners and specialist palliative home care teams, due to their complex medical conditions.
- The advantage of the interprofessional KOPAL approach did not show significant results regarding the reduction of hospital admissions.
- Identifying the palliative care needs of patients with advanced non-malignant chronic illnesses remains a challenge for healthcare providers.

## Introduction

In recent years, there has been a growing awareness of the palliative care needs of patients with advanced chronic non-malignant diseases, with their needs being comparable to those of cancer patients [1, 2]. The World Health Organization has identified dementia, chronic obstructive pulmonary disease (COPD) and congestive heart failure (CHF) among the chronic conditions requiring palliative care [3, 4], and there is evidence that palliative care improves outcomes in advanced stages of these conditions [5]. While care for these patients is often provided by general practitioners (GPs) alone [6–8], the complexity of their conditions, their high level of symptom burden, and the unpredictability of disease progression suggest the need for an interprofessional approach to care [9, 10]. In the last 6 months of life, patients with CHF or COPD often experience frequent hospitalisations, overuse of curative treatments, and increased admissions to intensive care [10, 11]. Similarly, patients with dementia are 3.1× more likely to be hospitalised in the last month of life [12]. Patients with advanced chronic non-malignant diseases could therefore particularly benefit from the timely involvement of a specialist palliative home care (SPHC) team in order to manage symptoms and prevent potentially aggressive interventions, including hospitalisation, intensive care and procedures, such as percutaneous endoscopic gastrostomy placement [13].

Despite increasing efforts to improve access, patients with advanced chronic non-malignant diseases constitute <10% of SPHC recipients [14]. The lack of interprofessional collaboration and uncertainty of illness trajectories are among the main barriers to the inclusion of these patients in SPHC [15–17]. While various interventions to promote interprofessional collaboration for cancer patients with palliative care needs have been investigated [18], approaches to address the palliative care needs of patients with advanced chronic non-malignant disease living at home remain scarce. In response to these challenges, a pilot study conducted in Australia investigated the impact of case conferences between palliative care specialists and GPs for patients with non-malignant diseases. Although based on a limited sample, the authors observed a substantial reduction in hospitalisations and emergency department visits among participants [19]. In light of these findings, our study aimed to implement case conferences between SPHC teams and GPs for patients with advanced chronic non-malignant diseases in the German healthcare system in order to achieve a shared care plan. By fostering interprofessional collaboration, we sought to reduce hospital admissions and enhance the overall quality of life of these patients.

## Methods

In the KOPAL study (‘Effectiveness of a concept for strengthening interprofessional collaboration for patients with palliative care needs’), we conducted a two-arm cluster-randomised controlled trial to evaluate the effect of a complex palliative care intervention compared with usual care. The intervention consisted of a structured SPHC nurse–patient consultation and an interprofessional telephone case conference with the patient’s GP, a SPHC physician and the SPHC nurse. The case conferences were scheduled for 30 minutes per patient. We refrained from specifying topics or roles, leaving the course, content and sequence of the case conferences to the participants. The previously developed KOPAL conversation guide provided the basis for the case conference and guided the nurse–patient consultation to focus on palliative care needs. SPHC teams received training on the use of the guide.

The primary objective of the intervention was to reduce hospital admissions within 48 weeks after baseline in patients with advanced heart failure, COPD and dementia. Detailed information on the study design and intervention can be found elsewhere [20]. However, due to restrictions imposed by the COVID-19 pandemic, the original study design had to be modified so that SPHC nurse–patient consultations were primarily telephone-based rather than face-to-face home visits. The KOPAL study was approved and confirmed to meet the ethical standards of the Declaration of Helsinki by the ethics committees of all participating centres.

## Recruitment

At first, we invited SPHC teams from northern Germany to participate in the study. After enrolling a SPHC team, all GP practices in their area of service that met the inclusion criteria (specialising in primary care or internal medicine, focusing on primary care and using computerised documentation software) were approached for participation. GPs who were currently active in a SPHC team were excluded from participation. Randomisation was performed at practice level using a web-based block randomisation application with stratification by study centre. Given the direct involvement of patients, providers and researchers in the intervention, blinding was not feasible.

In the next stage of recruitment, GPs screened their patients for eligibility using their computer software. Inclusion criteria encompassed the presence of one of the following diagnoses: (i) COPD with Global Initiative for Chronic Obstructive Lung Disease (GOLD) class 3–4, group D [21] or at least 1 hospitalisation due to severe exacerbation or at least 2 moderate exacerbations treated in outpatient care in the last 12 months, (ii) CHF with New York Heart Association (NYHA) class 3–4 [22] or at least 1 hospitalisation in the last 12 months and/or (iii) dementia with Global Deterioration Scale stage 4 or higher [23]. Patients were also required to have had one consultation with their GP within the previous 3 months and to be able to give both written and verbal consent. For patients with dementia, legal representatives signed the consent form on their behalf. Exclusion criteria were a cancer diagnosis within the last 5 years, current support from a SPHC team or current residency in a care facility.

### Sample size and power calculation

Due to recruitment challenges arising from the COVID-19 pandemic restrictions, the sample size was recalculated as detailed in the study protocol [20]. In an interim power calculation, we modified the initially planned sample size to better reflect the observed recruitment rates while maintaining scientific standards. In planning, 56 practices with 11 patients were assumed. However, at interim, 51 practices with 4 patients each better reflected ongoing recruitment, resulting in a design effect of 1.096 (intraclass correlation of 0.32). We modified the expected statistical power from 90% to 80% and assumed an expected reduction of hospitalisation rates between groups of 40% [24]. Further, assuming an overdispersion factor of 2, a dropout rate of 20% and a total sample size of 191 patients, it was considered adequate to achieve statistical significance with 80% power at a two-sided significance level of 5%.

### Data collection and assessment procedure

Data were collected between February 2020 and March 2022. Standardised interviews with patients, or proxies in the case of dementia, were conducted at all five time points (6, 12, 24 and 48 weeks after the baseline). The assessment included information on hospital admissions and sociodemographic characteristics. Patient self-rated health status was evaluated using a visual analogue scale (EQ-VAS), ranging from 0 (worst health status) to 100 (best possible health status). Health-related quality of life was assessed through the five-level version of the EuroQol 5D Scale (EQ-5D-5L), which covers domains, such as mobility, self-care, usual activities, pain or discomfort and anxiety or depression [25]. Based on the German value set for the EQ-5D-5L health status, an index value was determined using the mapping algorithm by van Hout *et al.* [26], with 0 representing death and 1 indicating perfect health with no problems in any dimension. Negative index values are possible, suggesting that health states are perceived as worse than death. Other secondary outcomes were symptom burden, palliative care needs, health status, advance directives, preferred place of death and the impact of the COVID-19 pandemic on health care [27]. Patients were given study diaries to record information, such as hospital stays, dates and reasons for admission, which served as a memory aid during the interviews. Additional information on patients’ medical history at baseline and their subsequent treatment was provided by GPs at 48 weeks or at the time of dropout, as well as information on hospital admissions 1 year before baseline and up to 48 weeks after the intervention. In cases of discrepancies between patient-reported and GP-reported admission or discharge dates, GP-reported records took precedence. Case conferences were observed by a non-participating member of the research team, and themes and interactions were documented using an observation protocol. The findings from the observations of the case conferences are reported elsewhere. Furthermore, we carried out an exploratory analysis of the collaboration status between GPs and SPHC teams at both baseline and the 48-week follow-up, including assessments for the study patients during the follow-up period. Given the potential emotional impact of using a guide focused on palliative care needs, we monitored suicidal ideation as a serious adverse event. Members of the research team and SPHC nurses were trained to immediately report any signs of a potentially dangerous clinical course, defined as imminent danger or severe neglect of medical care, directly to the principal investigator and to ensure that appropriate medical care was initiated. Given the high rates of hospitalisation and mortality in this patient population, which were unlikely to be causally related to the intervention [28], expedited reporting was not required for these outcomes. In the event of death, follow-up interviews with GPs were initiated, and the cause of death was assessed.

### Statistical analyses

The analysis followed the intention-to-treat principle. In the primary analysis, we examined the effect of the KOPAL intervention on the number of hospital admissions using a negative binomial regression model. The model included fixed effects for intervention, number of comorbidities and number of hospitalisations in the year prior to baseline. To account for differences in follow-up time between participants, logarithmic follow-up times were used as an offset in the analysis. As mortality rates in the sample were comparably low at 9.3% (*n* = 16), death was not modelled as a competing event. All recruited participants with at least one follow-up visit were included in the analysis. Incidence rate ratios (IRR) were calculated to compare the incidence of hospitalisations between both groups. This approach was repeated to assess the effect of the intervention on days spent in the hospital. In the secondary analysis, the effect on EQ-5D-5L and EQ-VAS scores was evaluated using linear mixed-effects models for repeated measures. The model included fixed effects for intervention and time, as well as the number of comorbidities and baseline scores. Random effects were included for individual patients and practices to account for within-patient and within-practice variability. To handle missing data in the secondary analysis, we used multivariate imputation by chained equations to impute missing values for both the EQ-5D-5L and EQ-VAS scales. Ten imputed datasets were generated and analysed, resulting in a pooled estimator. The R packages lme4, MASS and MICE were used for statistical analysis [29–31]. Descriptive statistics were used to assess the extent of collaboration between GPs and SPHC teams in both the intervention and control groups at baseline and 48-week follow-up.

## Results

### Sample description

**Figure 1:**
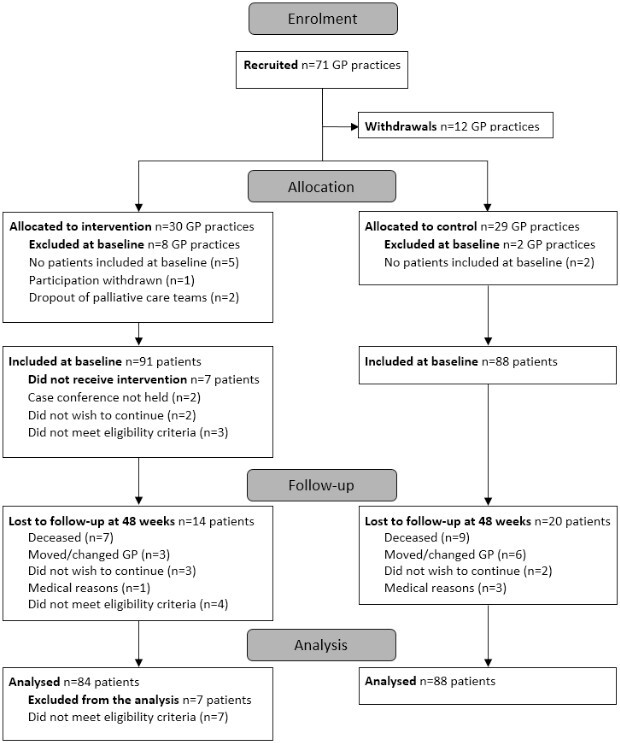
CONSORT Flow Chart.

The CONSORT chart (Figure 1) illustrates the flow of participants through the study. A total of 14 SPHC teams participated in the study, and from their areas of service, 71 GP practices were recruited and randomised. After dropout, the final sample consisted of 49 GP practices, with 22 practices in the intervention group and 27 practices in the control group. From these practices, 687 patients were identified as eligible and, hence, invited to participate by their GPs. Among them, 247 patients expressed interest in participating, and ultimately, 179 patients were included in the baseline assessment. In the intervention group, seven patients were later found ineligible: four due to a cancer diagnosis and three for not meeting the advanced stage of the inclusion diagnosis. Two patients did not receive the intervention as originally intended but were retained in the sample following the principle of intention-to-treat analysis. After accounting for eligibility and retention, the intervention group had 84 patients, while the control group had 88 patients available for statistical analysis.

The characteristics of the study sample are reported at the patient and GP level (see Tables 1 and 2). Participants in the intervention arm were slightly younger (mean age: 75.5 years) than in the control arm (mean age: 77.0 years). More men were enrolled in the intervention group (58.3%) than in the control group (51.1%), and most participants (64%) had primary or lower secondary school education. The groups had a similar distribution of the inclusion diagnoses of CHF, COPD and dementia, a comparable subjective health status (intervention: EQ-VAS score of 48.8, control: 48.0), and a mean number of comorbid conditions (intervention: 4.0; control: 3.9). The mortality rate during the 48-week follow-up period was 8.3% in the intervention group and 10.2% in the control group.

**Table 1:** Characteristics of the participants included in the primary analysis at baseline

Patient characteristics	Total (*n* = 172)	Intervention (*n* = 84)	Control (*n* = 88)
Mean age, yr (SD)	76.3 (9.8)	75.5 (9.8)	77.0 (9.9)
Gender, *n* (%)			
Women	78 (45.3)	35 (41.7)	43 (48.9)
Men	94 (54.7)	49 (58.3)	45 (51.1)
Marital status, *n* (%)			
Single	15 (8.7)	5 (6.0)	10 (11.4)
Married	97 (56.4)	47 (56.0)	50 (56.8)
Divorced	15 (8.7)	10 (11.9)	5 (5.7)
Widowed	45 (26.2)	22 (26.2)	23 (26.1)
Education, *n* (%)			
No formal education	3 (1.7)	1 (1.2)	2 (2.3)
Primary or lower secondary school education	110 (64.0)	55 (65.5)	55 (62.5)
Middle school education	32 (18.6)	17 (20.2)	15 (17.0)
Technical school certificate	10 (5.8)	6 (7.1)	4 (4.6)
High school diploma	17 (9.9)	5 (6.0)	12 (13.6)
Diagnosis of inclusion, *n* (%)			
CHF^*a^	81 (47.1)	39 (46.4)	42 (47.7)
COPD^*b^	68 (39.5)	33 (39.3)	35 (39.8)
Dementia	40 (23.3)	20 (23.8)	20 (22.7)
Mean no. of comorbidities (SD)	3.9 (2.0)	4.0 (2.0)	3.9 (2.1)
Subjective health^*c^ (SD)	48.4 (20.4)	48.8 (19.4)	48.0 (21.0)

The primary analysis includes 172 patients. *^a^CHF = Congestive heart failure. *^b^COPD = Chronic obstructive pulmonary disease. *^c^Subjective health measured via EQ-5D visual analogue scale.

**Table 2:** Characteristics of the general practitioners included in the primary analysis

GP characteristics	Total (*n* = 59)	Intervention (*n* = 27)	Control (*n* = 32)
Mean age, yr (range)	52.2 (35–72)	53.4 (38–64)	51.2 (35–72)
Gender, *n* (%)			
Women	34 (57.6)	15 (55.6)	19 (59.4)
Men	25 (42.4)	12 (44.4)	13 (40.6)
Form of practice, n^*^ (%)			
Individual practice	23 (46.9)	11 (50.0)	12 (44.4)
Group practice (shared patients and expenses)	20 (40.8)	7 (31.8)	13 (48.1)
Group practice (only shared expenses)	5 (10.2)	3 (13.6)	2 (7.4)
Medical care centres	1 (2.0)	1 (4.5)	0 (0.0)
Patients per quarter (range)	1665 (486–5040)	1786 (861–5040)	1556 (486–3400)

The primary analysis includes 59 participating GPs from 49 practices. ^*^n = 49.

The mean age of the participating GPs was 52.2 years (ranging from 35 to 72 years), and 57.6% were women. Among the 49 practices active in the study, 23 were individual practices, 20 were group practices with shared patient clientele and shared costs, 5 were group practices without shared patient clientele and 1 was a medical care centre. On average, the practices treated 1,665 patients per quarter (range: 486–5040 patients).

### Collaboration between GP and SPHC and follow-up communication

Data on collaboration (see Table 3) was gathered from 46 practices. In the intervention group, 17 practices (81%) had already established a working relationship with the SPHC team before the case conference, and two practices (9%) had previously worked with another SPHC team (9%). In the control group, 18 practices (72%) had worked with an SPHC team. Out of a total of 37 practices with SPHC collaborations, 19 collaborated for cancer patients only, 14 collaborated for both cancer and non-cancer patients and 4 were unable to provide information about the type of patients for whom they collaborated.

**Table 3:** Collaboration between GPs and SPHC teams in the sample

Group	Practices without existing collaboration with SPHC (%)	Practices with existing collaboration with SPHC (%)	Type of collaboration^*^
Intervention (*n* = 21)	2 (10%)	19 (90%)	Collaboration exclusively for cancer patients: 9
			Collaboration for cancer and non-cancer patients: 8
Control (*n* = 25)	7 (28%)	18 (72%)	Collaboration exclusively for cancer patients: 10
			Collaboration for both cancer and non-cancer patients: 6

^*^Missing data for two intervention and two control practices.

Regarding the follow-up communication between GPs, patients and SPHC teams, we collected data from 70 of the 84 initial patients in the intervention group. Of these, only 44 patients (63%) had a discussion with their GP about the results of the case conference, mainly initiated by the GP (34 out of 44). 37% of patients did not discuss the case conferences during the 48-week follow-up period. In four cases, the GP had further contact with the SPHC team following the case conferences, suggesting ongoing communication and collaboration. In addition, in two of these cases, there had been no collaboration with the SPHC team prior to the study. It is important to note, however, that these follow-up contacts did not always result in a referral. In total, four patients were referred to SPHC during the follow-up period, two in the intervention group and two in the control group.

### Effects of the intervention on hospitalisation

After controlling for the number of comorbidities and previous hospitalisation, the differences in hospitalisation in control versus intervention were not statistically significant (IRR 0.79, 95% CI: [0.49, 1.26], *P* = 0.31). Annualised hospitalisation rates were 0.96 (95% CI: [0.70, 1.30]) and 0.76 (95% CI: [0.54, 1.06]) for the control and intervention groups, respectively. For descriptive statistics of the outcome variables per baseline condition, see Supplementary File, table 1. When analysing the number of emergency admissions from all hospitalisations, there was also no significant effect of the intervention (IRR 0.79, 95% CI: [0.46, 1.35], *P* = 0.38). Similarly, there was no significant difference between the groups in the number of days spent in the hospital (IRR 0.65, 95% CI: [0.28, 1.49], *P* = 0.29). For detailed information on the incidence ratios for hospital admissions and total hospital days in the intervention and control arms, see Table 4.

**Table 4:** Incidence ratios for hospital admissions, emergency admissions and total hospital days in the intervention and control group

**Number of hospital admissions**							
	**1 year before baseline**		**48 weeks after baseline**				
	**Intervention, *n* (%)**	**Control,** ***n* (%)**	**Intervention, *n* (%)**	**Control, *n* (%)**	**IRR**	**95% CI**	***P***
0	42 (51.9)	50 (58.8)	47 (58.0)	45 (52.9)	0.79	0.49–1.26	0.31
1	19 (23.5)	19 (22.4)	21 (25.9)	19 (22.4)			
2	11 (13.6)	10 (11.8)	8 (9.9)	15 (17.6)			
3+	9 (11.1)	6 (7.1)	5 (6.2)	6 (7.1)			
**Number of emergency hospital admissions**							
	**1 year before baseline**		**48 weeks after baseline**				
	**Intervention, *n* (%)**	**Control,** ***n* (%)**	**Intervention, *n* (%)**	**Control, *n* (%)**	**IRR**	**95% CI**	***P***
0	49 (60.5)	54 (63.5)	53 (65.4)	52 (61.2)	0.79	0.46–1.35	0.38
1	20 (24.7)	21 (24.7)	18 (22.2)	17 (20.0)			
2	6 (7.4)	7 (8.2)	5 (6.2)	11 (12.9)			
3+	6 (7.4)	3 (3.5)	5 (6.2)	5 (5.9)			
**Number of days spent in hospital**							
	**1 year before baseline**		**48 weeks after baseline**				
	**Intervention, *n* (%)**	**Control,** ***n* (%)**	**Intervention, *n* (%)**	**Control, *n* (%)**	**IRR**	**95% CI**	***P***
0	42 (51.9)	50 (58.8)	47(58.0)	45 (52.9)	0.65	0.28–1.49	0.29
1–7	13 (16.0)	16 (18.8)	15 (18.5)	17 (20.0)			
8–14	11 (13.6)	5 (5.9)	8 (9.9)	9 (10.6)			
15–21	6 (7.4)	2 (2.4)	2 (2.5)	7 (8.2)			
22+	9 (11.1)	12 (14.1)	9 (11.1)	7 (8.2)			

IRR, incidence rate ratio; CI, confidence interval. Data available for *n* = 81 intervention patients and *n* = 85 control patients.

### Effects of the intervention on self-rated health and health-related quality of life

**Figure 2:**
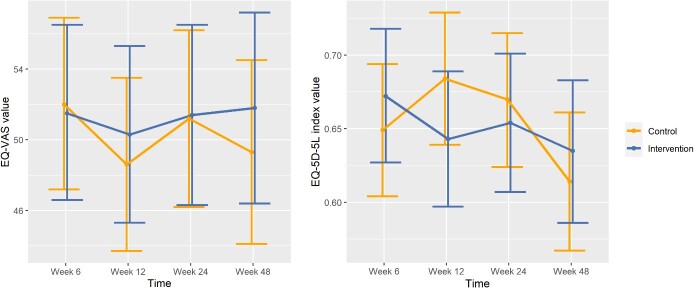
Effects of the KOPAL intervention self-rated health (EQ-VAS value) and health-related quality of life (EQ-5D-5L index value): Comparison of baseline-adjusted estimated marginal means with 95% confidence intervals by study arm.

The control group had a higher mean EQ-5D-5L index value at baseline (0.67) compared to the intervention group (0.59). This difference persisted at Week 48, with the control group’s mean index value at 0.66 and the intervention group’s mean index value at 0.61. Figure 2 displays the differences in baseline-adjusted marginal means between the intervention and control groups over time. The estimated difference in marginal means at Week 48 was −0.02 (95% CI: [−0.09, 0.05]), with a non-significant *P*-value of 0.53. Consequently, there is no significant difference in the EQ-5D-5L index value between the two groups at Week 48. By Week 48, the intervention group displayed a higher mean EQ-VAS score (52.1) in comparison to the control group (47.9). However, the non-significant *P*-value indicating the estimated difference in marginal means (*P* = 0.51) and the wide confidence interval (−9.95 to 4.99), encompassing 0, suggest that there is no statistically significant difference in the EQ-VAS score between the intervention and control groups once adjusted for baseline differences and comorbidities. Descriptive statistics of the outcome variables per each baseline condition can be found in the Supplementary File, tables 2-3. Throughout the trial, there was one case of suicidal ideation that was determined to be unrelated to the intervention. No harm arose from the intervention.

## Discussion

The KOPAL study aimed to improve the care of patients with progressive chronic non-malignant conditions living at home by establishing a timely interprofessional collaboration between GPs and SPHC teams. A complex intervention was developed that included a nurse–patient consultation focused on palliative care needs and an interprofessional telephone case conference with the patient’s GP, a SPHC physician and the SPHC nurse. However, quantitative analysis did not show the hypothesised significant reduction in hospital admissions or hospital days at the end of the 48-week follow-up period. There were also no significant effects on the secondary outcomes of subjective health status and health-related quality of life.

### Comparison with existing literature

The KOPAL intervention provided a low-threshold form of collaboration for GPs and SPHC teams by using single telephone case conferences. A similar approach was used in a randomised controlled trial by Mitchell *et al*. (2008) involving patients already referred to palliative care, both with and without cancer. They encouraged GPs to participate in teleconferences with specialist palliative care teams to develop treatment plans. Ultimately, they found no statistically significant reduction in quality of life. In a subsequent exploratory pilot study, Mitchell *et al.* (2014) extended this approach by conducting case conferences with prior assessment of palliative care needs for 23 patients with end-stage heart failure and lung disease. The results showed a trend towards reduced healthcare utilisation, although there was a high level of statistical uncertainty due to the small sample size. Rather than relying solely on the stage of the disease, they identified patients by asking providers if they would be surprised if the patients died within the next 12 months. These preliminary findings suggest that palliative care case conferences are promising for this group of patients, but that the timing of the intervention may be critical to its success [19, 32].

While research has been investigating the early versus timely onset of palliative care, there is large evidence for the benefit of palliative care services [33]. Nevertheless, studies with interventions focusing on integrating non-cancer patients into SPHC have been scarce. Brumley *et al.* (2007) reported significant reductions in hospital days and emergency department visits, as well as improvements in patient satisfaction. However, their study encompassed a complex multi-modal intervention [34], which may suggest that the one-time KOPAL intervention may not have been appropriate to meet the complex needs of the patients, thereby limiting its overall impact on their care. Another study applied a hospital-based approach to the early integration of palliative care patients with progressive chronic non-malignant conditions and reported a significant reduction in hospitalisation as well as re-hospitalisation and intensive care admissions [35].

Although studies have shown a high level of openness among GPs to consult with SPHC teams and a willingness to engage in shared care [36], initiating SPHC access for these patients proves to be a challenge for GPs, SPHC teams, as well as the patients. A recent meta-analysis by Chyr *et al.* (2022) found little to no effect in studies aiming at integrating ambulatory palliative care into the chronic care of patients with chronic non-malignant conditions on symptom burden and health-related quality of life [37], foremost due to the difficulties in identifying patients in need of palliative care [38, 39]. Despite the strict inclusion criteria for advanced disease, our results suggest that a substantial proportion of patients in the trial may not have been at a stage of disease where SPHC was needed. This may explain why the intervention did not significantly reduce hospital admissions or improve patients’ quality of life. It remains unclear whether this is due to the diversity of the diseases, the assessment of disease progression by the GP, or whether the inclusion criteria should reflect the need for palliative care rather than the progression of the disease.

### Strengths and limitations

To the best of our knowledge, this study is the first to examine the impact of telephone case conferences between GPs and SPHC teams on the care of patients with progressive non-malignant chronic conditions living at home in a European context. By focusing specifically on this patient population, the study addressed an important yet understudied area of advanced chronic diseases. One of the main limitations is related to the changes in study procedures caused by the pandemic and the resulting recruitment difficulties within the funding period. This significantly reduced the number of cases, affecting the study’s statistical power [20].

The results of our study may also have been influenced by the selection of the sample and the design of the intervention. The qualitative analyses, which will be discussed in more detail elsewhere, showed that the health care providers involved were willing and motivated to improve interprofessional collaboration, but a number of barriers to achieving this were identified. While the value of case conferences was recognised by the participating professions, especially in identifying psychosocial and nursing needs and inadequate emergency and advance care planning, they criticised the lack of sustainability of this one-off intervention. Given the significant proportion of patients who did not engage in discussions following the case conferences, more specific guidance on the content of case conferences or more systematic implementation of their outcomes, as demonstrated by Mitchell *et al.* (2014), may have been beneficial. Changing home consultations to telephone consultations between patients and SPHC nurses for the majority of cases, due to restrictions imposed by the pandemic, may also have impacted the effectiveness of the intervention. It also remains unclear to what extent the pandemic affected hospital admissions in our target group, who were in the high-risk group, and where hospital admissions were prevented.

Additionally, there is a potential for selection bias, as GP practices interested in palliative care may have been more inclined to participate, introducing a positive bias in the quality of palliative care provision. Given the high percentage of GPs already collaborating with SPHC teams on our study, which is comparable to the German average of 83.7%, assessing the impact of new exposure to SPHC through case conferences was not feasible in our study [40].

### Implications for research and practice

Our findings underscore the importance of conducting further research and refining interventions towards the timely and effective integration of SPHC into the care of patients with progressive chronic non-malignant conditions living at home. Increasing the sample size in future studies will be essential to bolstering the validity and applicability of the results. To gain a better understanding of the reasons behind the limited impact of the intervention on collaboration, we also conducted a qualitative study involving in-depth interviews with participating GPs, SPHC teams, patients and their family members, as well as analysing the content of the case conferences.

Future studies should also develop structures for conducting case conferences and translating their findings back into practice. Previous research indicated that timing is critical to the effectiveness of case conferences between GPs and specialist palliative care services [41]. As SPHC services rely on GP prescriptions, there is a need for increased sensitivity among GPs in determining the appropriate time to initiate these services. While some approaches have been developed for chronic non-malignant conditions, such as the RADboud indicators for palliative care needs or the Supportive and Palliative Care Indicators Tool (SPICT), there are few studies that have examined the actual impact of these on patient care and outcomes [42, 43]. Refining the timing of interventions should therefore be the focus of future research.

## Conclusion

The overall objective of the KOPAL study was to facilitate joint palliative care planning and the timely transfer of patients with advanced chronic non-malignant conditions to SPHC. The results of the study did not show a statistically significant benefit of the intervention on hospitalisation rates or patients’ quality of life compared to usual primary care. Only for a limited number of patients was there further collaboration between the GP and the SPHC team, highlighting the need to identify patients who could benefit from palliative case conferences according to a needs-based approach rather than according to the progression of the disease. In this way, the results of the KOPAL study provide valuable guidance for the design of subsequent research on collaboration between GPs and palliative care providers. The utilisation of SPHC services by GPs has seen a major increase in recent years, particularly for patients suffering from cancer [44–46]. However, in light of the increasing global ageing population and the rise of chronic conditions, addressing the needs of patients with advanced chronic conditions will require the development of strategies to overcome barriers and improve collaboration between various healthcare providers to close gaps in care.

## Supplementary Material

aa-23-2293-File002_afae100

## Data management and sharing

De-identified data will be available upon reasonable request ending 7 years after this publication. Proposals should be sent to Gabriella Marx (g.marx@uke).

## Trial registration

The trial was prospectively registered with the German clinical trial register under the identification number DRKS00017795.
