# Supplementary material for: Evaluating palliative care case conferences in primary care for patients with advanced non-malignant chronic conditions: a cluster-randomised controlled trial (KOPAL)
Source: Age Ageing. 2024 May 23;53(5):afae100. doi: 10.1093/ageing/afae100 (PMC11116821; doi:10.1093/ageing/afae100)
Supplement: aa-23-2293-File002_afae100 [file aa-23-2293-file002_afae100.docx]

**Supplementary Material**

**Table 1** Annualised number of hospital admissions at baseline and 48-week follow-up by inclusion diagnosis

| **Diagnosis** | **N** | **Mean number of hospital admissions (SE)** | | | |
| --- | --- | --- | --- | --- | --- |
|  |  | **1 year before baseline** | | **Follow-up at 48 weeks** | |
|  |  | **Intervention** | **Control** | **Intervention** | **Control** |
| CHF | 78 | 0.89 (0.21) | 0.73 (0.17) | 1.04 (0.25) | 1.29 (0.28) |
| COPD | 63 | 1.17 (0.27) | 0.67 (0.18) | 0.72 (0.22) | 1.09 (0.28) |
| Dementia | 40 | 0.70 (0.23) | 0.95 (0.29) | 0.57 (0.23) | 0.95 (0.33) |

**Table 2** Mean subjective health status measured via EQ-VAS at baseline and 48-week follow-up by inclusion diagnosis

| **Diagnosis** | **N** | **Mean subjective health status (SE)** | | | |
| --- | --- | --- | --- | --- | --- |
|  |  | **Baseline** | | **Follow-up at 48 weeks** | |
|  |  | **Intervention** | **Control** | **Intervention** | **Control** |
| CHF | 81 | 51.25 (3.49) | 50.95 (3.37) | 56.46 (3.95) | 50.63 (3.77) |
| COPD | 68 | 44.09 (3.78) | 47.09 (3.67) | 47.90 (4.41) | 48.82 (3.95) |
| Dementia | 40 | 46.25 (4.89) | 40.75 (4.89) | 48.18 (5.56) | 39.52 (5.72) |

**Table 3** Mean quality of life measured via EQ-5D-5L index value at baseline and 48-week follow-up by inclusion diagnosis

| **Diagnosis** | **N** | **Mean quality of life (SE)** | | | |
| --- | --- | --- | --- | --- | --- |
|  |  | **Baseline** | | **Follow-up at 48 weeks** | |
|  |  | **Intervention** | **Control** | **Intervention** | **Control** |
| CHF | 81 | 0.61 (0.04) | 0.69 (0.04) | 0.63 (0.05) | 0.65 (0.04) |
| COPD | 68 | 0.56 (0.05) | 0.71 (0.05) | 0.57 (0.05) | 0.73 (0.05) |
| Dementia | 40 | 0.53 (0.06) | 0.52 (0.06) | 0.45 (0.06) | 0.39 (0.06) |
